# 37 kDa LRP::FLAG enhances telomerase activity and reduces senescent markers *in vitro*

**DOI:** 10.18632/oncotarget.21278

**Published:** 2017-09-27

**Authors:** Tyrone C. Otgaar, Eloise Ferreira, Sibusiso Malindisa, Martin Bernert, Boitelo T. Letsolo, Stefan F.T. Weiss

**Affiliations:** ^1^ School of Molecular and Cell Biology, University of the Witwatersrand, Wits 2050, Republic of South Africa; ^2^ Present Address: Department of Life and Consumer Sciences, University of South Africa, Florida 1710, Republic of South Africa

**Keywords:** aging, LRP/LR, LRP::FLAG, telomerase, telomeres

## Abstract

One of the core regulators of cellular aging are telomeres, repetitive DNA sequences at the ends of chromosomes that are maintained by the ribonucleoprotein DNA polymerase complex, telomerase. Recently, we demonstrated that knockdown of the 37kDa/ 67kDa laminin receptor (LRP/LR), a protein that promotes cell viability in tumorigenic and normal cells, reduces telomerase activity. We therefore hypothesized that upregulating LRP/LR might increase telomerase activity and impede aging. Here we show that overexpression of LRP::FLAG resulted in significantly elevated hTERT levels, telomerase activity and telomere length, respectively, with concomitantly reduced levels of senescence markers. These data suggest a novel function of LRP/LR hampering the onset of senescence through elevating hTERT levels and telomerase activity, respectively. LRP::FLAG might therefore act as a potential novel anti-aging drug through the impediment of the cellular aging process.

## INTRODUCTION

Aging is a degenerative process characterized by the accumulation of detrimental changes that cause deterioration of the physiological functioning of the organism [1]. The process is characterized in cells by telomere erosion, genomic instability, mitochondrial dysfunction and stem cell exhaustion [1]. These stresses drive cellular senescence by limiting the replicative and regenerative potential of cells, this ultimately drives functional decline and disrupts tissue homeostasis to accelerate the aging process [2, 3]. One of the core regulators of human cellular aging are telomeres, repeated elements of TTAGGG that “cap” chromosome ends [4, 5]. Age-dependent loss of telomere functions, due to the “end replication” problem of DNA polymerases, telomere fusions and insufficient telomerase activity, eventually causes cell cycle arrest to induce a senescent state [4-8]. To circumvent aging, most epithelial and tumorigenic cells re-activate the enzyme telomerase, which can lead to telomere extension [9, 10]. This telomeric extension provided by telomerase maintains and elongates telomere length to effectively increase a cell’s replicative capacity [9-11]. Interestingly, there have been a number of findings illustrating that in addition to its role in telomere extension, telomerase and its enzymatic subunit, telomerase reverse transcriptase (TERT), have other functions. These additional functions, otherwise known as extra-telomeric functions, all coincide to preserve cell viability [12]. A few of these include: DNA repair, cell proliferation, mtDNA protection as well as influencing a number of cellular pathways offering pro-proliferative functions [12, 13]. TERT’s numerous roles can be attributed to its ability to interact directly or indirectly with various cellular proteins. One such protein, that we have proven to interact with TERT/telomerase, is the 37kDa/ 67kDa laminin receptor (LRP/LR) [14]. LRP/LR is a non-integrin transmembrane protein located predominantly on the cell surface, and to a lesser extent intracellularly in the cytosol, peri-nuclear as well as nuclear domains [14, 15]. The wide-spread localization of LRP/LR as well as its numerous interactions shared with extracellular matrix proteins allows its key role in multiple processes such as: cell growth, adhesion, differentiation, migration, translational processes and maintenance of nuclear structures required for survival regimens [14-21]. The multi-functionality of LRP/LR includes vital roles in diseases such as cancer [14, 16-21], prion diseases [22, 23] and Alzheimer’s disease [24]. Recently, we demonstrated that knockdown of LRP/LR, reduces telomerase activity in tumorigenic and normal cells [14, 15]. This suggests that LRP/LR, which promotes cell viability, plays a pivotal role in cancer processes via influencing telomerase activity [14]. Considering that telomerase and telomeres play antagonistic roles in cancer and aging [25], we postulated that an upregulation of LRP/LR may increase telomerase activity and possibly impede the aging process.

## RESULTS

**Figure 1:**
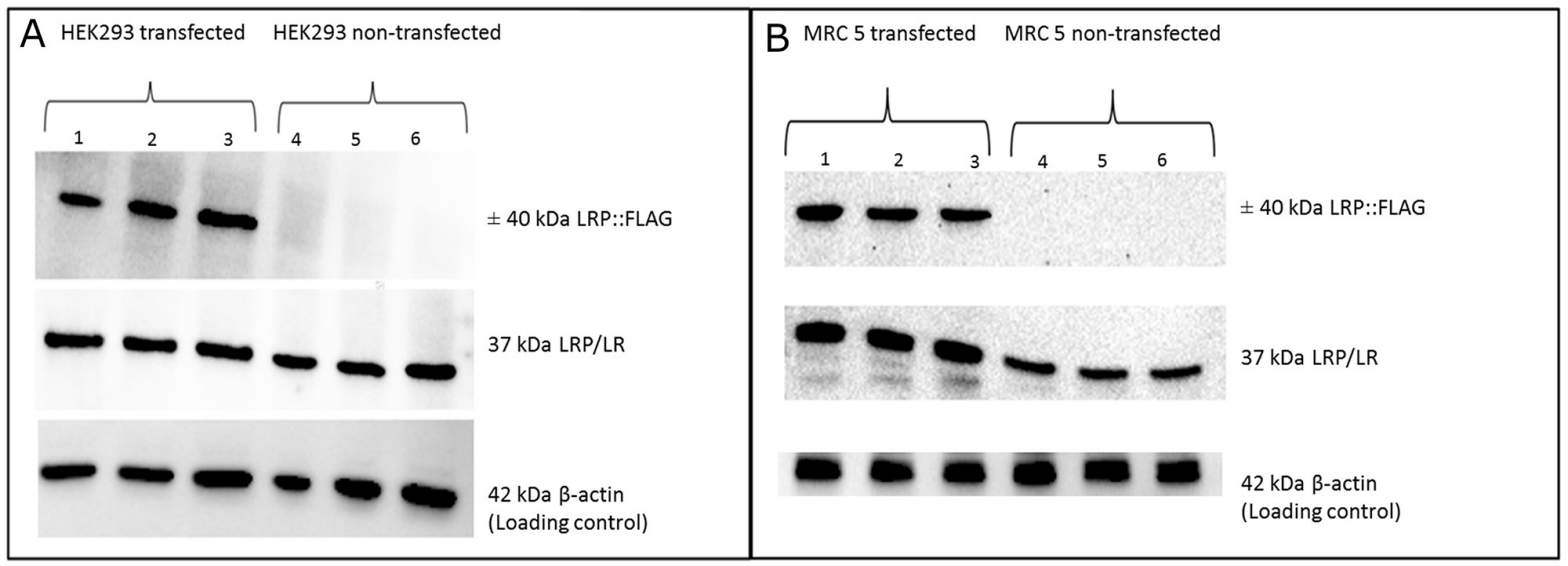
Overexpression of LRP::FLAG in HEK293 and MRC5 cells **(A, B)** The expression of the LRP:FLAG protein was determined for both HEK293 and MRC 5 cells transfected with the pCIneo-LRP-FLAG construct (lane 1-3) as well as non-transfected HEK293 and MRC 5 cell samples (lane 4-6). Analysis revealed that LRP::FLAG was found to only be expressed in HEK293 and MRC 5 transfected cell samples, with no LRP::FLAG detection in the non-transfected cell samples.

**Figure 2:**
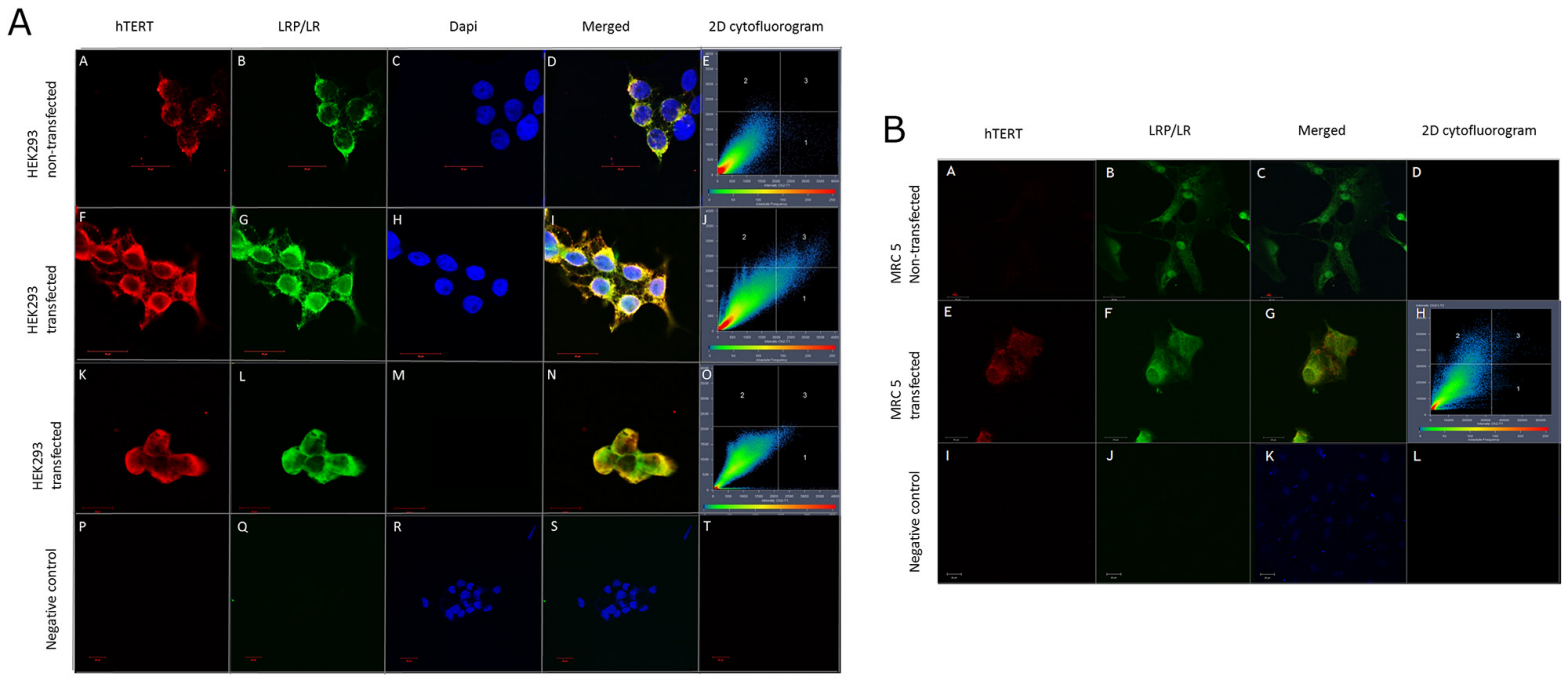
LRP and LRP::FLAG co-localizes with hTERT **(A, B)** Intracellular localization and co-localization patterns of hTERT and LRP/LR and hTERT and LRP::FLAG in transfected as well as non-transfected HEK293 and MRC 5 cells. hTERT was detected with anti-hTERT primary and APC coupled secondary antibodies (A-A, A-F, A-K, B-A, B-E); LRP/LR was detected with anti-LRP/LR IgG1-iS18 primary and FITC coupled secondary antibodies (A-B, A-G, B-B, B-F); LRP::FLAG detected with anti-FLAG and FITC coupled secondary antibodies (A-L); Dapi nuclear staining (A-C, A-H, A-M, A-R); merge between LRP/LR and hTERT (A-D, A-I, B-C, B-G) and LRP::FLAG and hTERT (A-N) illustrates co-localization caused by the spatial overlap between the proteins. 2D Cytoflurograms confirmed that the overlap observed was co-localization between the proteins (A-E, A-J, A-O, B-H). Secondary antibody controls confirmed no auto-fluorescence or non-specific binding (A-P, A-Q, A-R, A-S, A-T, B-I, B-J, B-K, B-L). Images were taken at 630x and negative controls were obtained at 400x magnification. All scale bars are 20μm.

**Figure 3:**
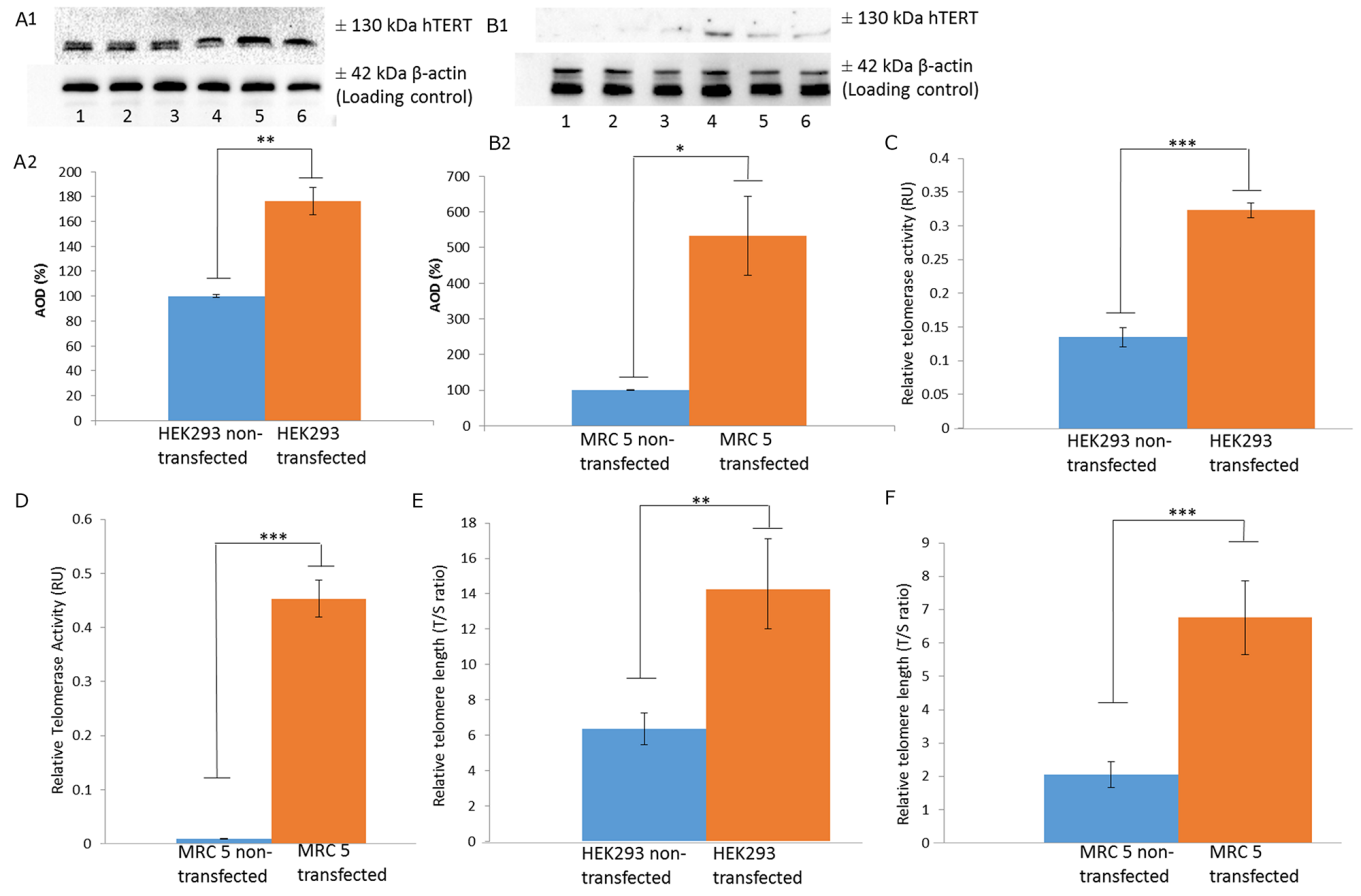
LRP::FLAG overexpression significantly elevates hTERT levels, telomerase activity and telomere length **(A-B)**, Western blot of hTERT levels in non- transfected (lane 1-3), and transfected (lane 4-6) HEK293 cells (A) as well as non-transfected (lane 1-3), and transfected (lane 4-6) MRC 5 cells (B). Densitometric analysis of hTERT expressed as a relative percentage in change respective to the non-transfected samples. **(C-D)** Relative telomerase activity (RU=relative units) in non-transfected and transfected HEK293 (C) as well as MRC 5 (D) cells shown in Log concentration (Log amole). **(E-F)**, Relative telomere length in transfected and non-transfected HEK293 **(E)** as well as MRC 5 **(F)** lines as a telomere/36B4 (T/S) ratio to illustrate mean telomere length between samples. Sample size n= 3 biological repeats per cell line. Data as a mean ±sd. *p<0.05, **p<0.01, ***p<0.001 by paired *t*-test.

**Figure 4:**
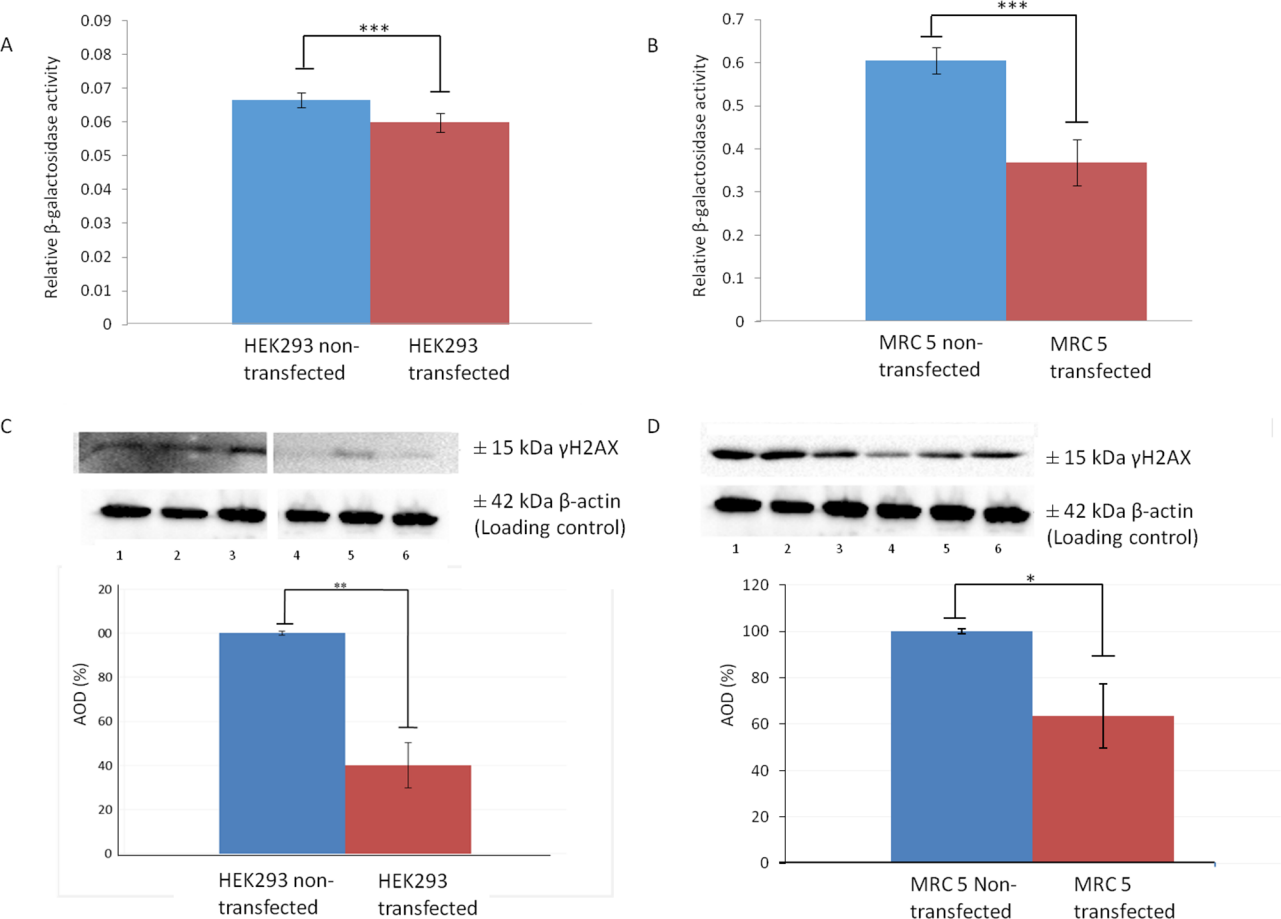
LRP::FLAG overexpression significantly decreases the levels of senescent markers **(A-B)** Change in enzymatic activity of β-galactosidase following LRP::FLAG overexpression in HEK293 (A) and MRC 5 (B) cells when compared to the non-transfected cell lines. **(C, D)** Densitometric analysis of γH2AX levels expressed as a percentage change with non-transfected (lanes 1-3) compared to transfected lines (lanes 4- 6) for both HEK293 **(C)** and MRC 5 **(D)**. Sample size n= 3 biological repeats per cell line. Data as a mean ±sd. *p<0.05, **p<0.01, ***p<0.001 by paired *t*-test.

In order to validate that upregulation of LRP/LR does in fact increase telomerase activity, non-tumorigenic MRC 5 cells, at population doubling 23, and HEK293 cells were stably transfected with a pCIneo-moLRP-FLAG plasmid effectively elevating LRP::FLAG levels within the cells (Figure 1A, 1B). Western blotting was performed to confirm the presence of the LRP::FLAG protein and therefore stable transfection of the cells and to further assess LRP levels (Figure 1A, 1B). Upon confirming LRP::FLAG overexpression we proceeded to evaluate the change in expression patterns of LRP and hTERT following transfection. We previously observed co-localization between LRP and hTERT within MDA-MB231 and HEK293 cells [14], however, expression patterns and co-localization between these two proteins have not been confirmed in somatic cells with low/ undetectable levels of TERT. Immunofluorescence microscopy was employed to confirm the co-localization between the proteins in both MRC 5 and HEK293 transfected as well as non-transfected cell lines. The merged panels in Figure 2A, 2B, represent a merge of the red (hTERT) and green (LRP/LR and LRP::FLAG), where areas of yellow fluorescence indicated spatial overlap and possible interaction of the two proteins. The intracellular co-localization of LRP/LR and hTERT was pronounced for both non-transfected and transfected HEK293 cells (Figure 2A). Interestingly, the transfected MRC 5 cells displayed a striking increase in TERT fluorescence and co-localization (between LRP/LR and hTERT) when compared to the non-transfected MRC 5 cells which displayed negligible fluorescence for hTERT and no detectable co-localization (Figure 2B). Since co-localization between hTERT and LRP/LR was observed, we wanted to evaluate whether this pattern was also shared between hTERT and LRP::FLAG. To this end only HEK293 cells were utilized, where we found that LRP::FLAG behaved in a similar fashion to endogenous LRP/LR in localization and also co-localized with hTERT (Figure 2A panels K-N). This further suggested that the plasmid derived protein was correctly post-translationally processed, and was able to perform similar processes and interactions as its endogenous counterpart. The merged images and 2D-Cytoflurograms further revealed that a greater degree of co-localization occurred in cells overexpressing LRP::FLAG (Figure 2A, 2B). The increased hTERT levels post pCIneo-moLRP-FLAG transfection therefore encouraged us to further investigate telomere and telomerase dynamics. Telomere biology is one of the core regulatory mechanisms for the aging process in humans [4, 9] and is therefore a key aspect to assess in senescence and aging. A main factor involved in regulating telomere attrition is insufficient maintenance by telomerase; which in turn is limited by the expression of its catalytic subunit, hTERT [9, 26]. We thus investigated whether overexpression of LRP::FLAG influences hTERT-levels. Interestingly, western blot and subsequent densitometric analysis revealed a significant increase in hTERT levels in pCIneo-moLRP-FLAG transfected versus non-transfected HEK293 and MRC 5 cells (Figure 3A, 3B). HEK293 cells overexpressing LRP::FLAG revealed a 176,4% (n= 3, p= 0.0014, *t* test) increase in hTERT levels, while MRC 5 cells overexpressing LRP::FLAG revealed a 533.11% increase (n= 3, p= 0.0312, *t* test) in hTERT levels. The elevation of hTERT levels via LRP::FLAG overexpression in MRC 5 cells with little or no endogenous hTERT expression [27], indicates that LRP::FLAG enhances hTERT levels. These findings led us to determine whether LRP::FLAG mediated elevated levels of hTERT may subsequently affect the activity of telomerase, a ribonucleo-protein, acting as a key component to counteract telomere-dependent senescence by maintaining telomere length [7, 9]. Telomerase activity was detemined with the TRAPeze RT telomerase detection kit (Merck Millipore) via real time qPCR. HEK293 cells overexpressing LRP::FLAG revealed a 2.937 fold increase (n=4, p=2.91*10^-5^, *t* test) in telomerase activity compared to the non-transfected cells (Figure 3C, 3D). LRP::FLAG overexpression in MRC 5 cells revealed a 52.195 fold increase (n=4, p=2.38*10^-5^, *t* test) in telomerase activity compared to non-transfected cells with minimal telomerase activity (Figure 3C, 3D). In order to investigate whether the LRP::FLAG mediated increased telomerase activity results in an elongation and maintenance effect of the telomere ends, qPCR was utilized and the data analyzed according to Cawthon et al., (2002) using *[2*^*ΔCT (telomeres)*^*/2*^*ΔCT (36B4)*^*]* [28]. Prior to telomere length analysis, the reference gene, acidic ribosomal phosphoprotein (36B4), was analyzed to ensure equal DNA content between transfected and normal cell lines (Supplementary Figure 1A, 1B) [28]. A significant difference in telomere length was detected for both HEK293 and MRC 5 cells overexpressing LRP::FLAG (Figure 4E, 4F). Transfected HEK293 cells displayed a 2.236 fold increase (n= 4, p= 0.001909, *t* test) and transfected MRC 5 cells at population doubling 40 displayed a 2.839 fold increase (n= 4, p= 0.0002, *t* test) in mean telomere length, compared to their respective non-transfected cell lines. Since telomerase plays a role in cellular senescence and aging, these results regarding telomere dynamics (hTERT level, telomere length and telomerase activity) encouraged us to investigate whether LRP::FLAG may play a role in the senescence process. We therefore assessed the production and accumulation of specific senescence markers in response to LRP::FLAG expression. We selected β-galactosidase activity as our primary senescence marker as this enzyme is influenced by telomere dysfunction and accumulates as cells age or reach senescence [29, 30]. Furthermore, the use of this enzyme in conjunction with an additional marker is broadly utilized to track cellular aging [29, 30]. Transfected and non-transfected cell lines were allowed to undergo a minimum of 20 population doublings before this marker was assessed. To evaluate the enzyme’s activity in both transfected and non-transfected cells; cell lysates were incubated with an assay buffer containing ortho-Nitrophenyl-β-galactoside at pH 6 (reporter lysis β-galactosidase assay, Promega), which when reduced allows for a quantitative measurement of β-galactosidase [29]. In fact, Lee et al., (2006) illustrated that senescence associated or lysosomal β-galactosidase can be detected if incubated for an extended period of 12 hours. HEK293 cells overexpressing LRP::FLAG showed a significant 1.111 fold (10%) reduction (n=3, p= 4,22E-05, *t* test) in β-galactosidase activity, when compared to non-transfected cells (Figure 4A), whereas MRC 5 fibroblasts revealed a significant 1.638 fold (40%) reduction in β-galactosidase activity (n= 3, p= 0.0008, *t* test) after LRP::FLAG overexpression (Figure 4B). To further substantiate this impediment of the aging process we assessed the levels of an additional senescent marker; γH2AX foci. These foci are histones that are specifically phosphorylated at pSer139 and serve to mark sites of DNA damage as well as double stranded breaks which accumulate with increased cellular age due to the loss of telomeric ends [31, 32]. Overexpression of LRP::FLAG caused a significant decrease in the levels of γH2AX in both cell lines (Figure 4C, 4D). HEK293 cells overexpressing LRP::FLAG exhibited a 60.78% (n= 3, p= 0.0017, *t* test) reduction in γH2AX levels, while MRC 5 cells overexpressing LRP::FLAG displayed a significant 40% (n= 3, p= 0.009, *t* test) decrease in total γH2AX levels. Although, a reduction in both senescence markers was observed in the HEK293 cells it must be noted that these levels were exceptionally low (basal levels) and may in fact be due to extensive sub-culturing or other relevant stresses. In addition, basal levels of these markers have been previously observed in exceptionally low amounts [33, 34].

## DISCUSSION

Previous studies have shown LRP/LR and telomerase to play critical roles in carcinogenesis [14, 15, 35, 36]. In addition, it has been found in most cancer types that LRP/LR, hTERT and telomerase are overexpressed to promote cell viability as well as to retain an immortalized state [14, 15, 35, 36]. Moreover, telomerase up-regulation is a necessary requirement for cells to bypass apoptotic and senescence pathways during critical telomere shortening in order to become immortalized or possibly tumorigenic [37, 38]. Recently, we have shown that LRP/LR not only co-localized, but in fact, interacted with hTERT; the catalytic subunit of telomerase via a co-immunoprecipitation assay [14]. In addition, knockdown of LRP/LR reduced telomerase activity in tumorigenic and non-tumorigenic cells [14]. Our hypothesis was further based on the evidence that whilst non-tumorigenic somatic cells senesce due to telomere attrition, introduction of hTERT at any stage of the cell cycle is sufficient to immortalize cells without additional risk of tumorigenic onset [9, 26, 39]. We have shown that aside from tumorigenic and other highly proliferative cell lines that naturally transcribe TERT; a functional relationship between LRP/LR and hTERT exists in somatic cells such as fibroblasts, which are only capable of displaying such an interaction provided TERT production is stimulated. Interestingly, this interaction appears to be localized to the nucleus, peri-nuclear compartments and cytosol. This distribution of TERT, while uncommon, has been shown in studies where shuttling between the nucleus and cytoplasm is imperative for cell immortalization in both tumorigenic and non-tumorigenic cell lines [40-42]. In support of this, a growing amount of data on extra-telomeric functions of TERT has illustrated its cytoplasmic distribution especially for mitochondrial function and protection [40-42]. Using confocal microscopy we have identified that LRP/LR interacts with TERT, where this relationship is further enhanced by the elevation of LRP/LR levels, such as overexpression of the LRP::FLAG protein. In fact, it was found that upon overexpression of LRP::FLAG that the levels of TERT and co-localization between TERT and LRP/LR increased. Our results suggest that LRP/LR shares an interaction/association with hTERT, which was previously reported in HEK293 and metastatic breast cancer cells (MDA-MB231) [14]. The confirmation of such an interaction between LRP/LR and hTERT indicates that targeting LRP/LR could be utilized to alter the functioning of hTERT or telomerase to influence either cancer or aging [14, 15]. LRP::FLAG may also play a direct or indirect role in promoting endogenous hTERT levels [14, 15]. These findings suggest that LRP/LR, aside from interacting with TERT, may play a role in the endogenous production of TERT through translational or transcriptional processes. Additionally, the presence of LRP::FLAG may contribute to these functions and processes performed by endogenous LRP. One such function is its interaction with the ribosomal complex where it facilitates translational processing [43]. Alternatively, LRP could carry out this unique function of elevating TERT levels by interaction with the nuclear envelope to allow chromatin remodeling to subsequently expose the TERT gene leading to increased transcription [44]. The association of these two proteins as well as the elevation in TERT post LRP::FLAG overexpression appears to positively impact on telomerase dynamics and telomere homeostasis. This can be noted due to the significant elevation in telomerase activity accompanying the increased TERT levels. This correlates with the previous finding that telomerase activator (TA65) elevates TERT levels with a corresponding increase in mean telomere length [45]. Our findings confirm the dogma in telomere biology: the introduction of hTERT or the externally mediated elevation of hTERT levels increases telomerase activity in a non-oncogenic fashion; a common phenomenon in many immortalized cell lines [9, 10, 26, 27, 39, 45]. It must be noted that post transfection with the pCIneo-moLRP-FLAG plasmid there was no apparent phenotypic or morphological alterations indicative of transformed cells (such as loss of contact inhibition or growth in low serum) that may have accounted for elevation in telomerase activity [9]. The telomerase activity described here refers to its core elongating function of attaching telomeric repeats to the terminal ends of chromosomes, and thus enhancing the cell’s proliferative potential [10, 35, 46] and preventing cellular senescence [9, 32]. Therefore it appears that LRP::FLAG overexpression aids in increasing telomerase activity sufficiently to increase proliferative potential and inhibit the activation of senescence associated pathways. Aside from increasing hTERT levels, we suggest that LRP/LR and/or LRP::FLAG may play a role in promoting the association of the hTERC and hTERT subunits to form the active telomerase based on the fact that telomerase activity appears to be regulated by LRP levels in both cancer and normal cells where both proteins have been shown to co-localize and interact [14]. In addition, the large difference in telomere length between transfected and non-transfected lines is a clear indicator that telomere length was indeed maintained. This suggests that the increased telomerase activity following the production of LRP::FLAG is sufficient to maintain telomere length and prevent the accumulation of short telomeres, a characteristic of telomere-dependent senescence [9, 10, 46]. This elongation allows cells to reverse and impede the aging process, and essentially immortalize themselves. Alternatively, the difference in telomere length post transfection may indicate a re-population by cells with longer telomeres [10, 47]. The reduction of both molecular aging markers further substantiates that the telomere replenishment occurring is in fact impeding senescence. More specifically the reduction of β-galactosidase, aside from suggesting an impediment to the senescence process also suggests that telomere dysfunction is not occurring [30, 48]. The accumulation of short/damaged telomeres with increased replicative stress and age is believed to be one of the major causatives of DNA damage associated with aging [10, 49]. This damage further accelerates the loss of regenerative capacity in organisms [10, 49]. The significant decrease in γH2AX concomitantly with the increase in telomere length therefore indicates that the overexpression of LRP::FLAG provides an efficient means of reducing the effects of replicative stress by eliminating or preventing the accumulation of DNA damage [32, 46, 49]. TERT has previously been found to prevent the accumulation of DNA damage or mediate in the repair process to decrease the amount of foci present [12, 49, 50] further suggesting that the increased TERT present may be performing extra-telomeric functions in addition to telomere maintenance [12, 50]. Alternatively, LRP may have a role in DNA protection or in the repair process itself, however the exact nature and function still needs to be assessed. One possible mechanism that may explain this is that LRP/LR is known to play a role in supporting nuclear structure and overexpression of LRP::FLAG may thus provide an additional function of increasing nuclear integrity [15, 44]. This would essentially maintain nuclear shape which could alleviate and prevent additional DNA damage. Collectively, these findings strongly suggest that overexpression of LRP::FLAG in non-tumorigenic cell lines is sufficient to induce a physiological change. This change appears to allow the state and processing of young cells to be retained, by either slowing down or impeding the aging process [10, 32, 39]. In this regard, towards the end of our study the non-transfected MRC 5 cells underwent senescence at around passage doubling 49-50, while the transfected MRC 5 cells underwent an additional 20 passage doublings before the end of the study. Retention of pre-senescent functioning in normal cells holds important implications in the medical and pharmaceutical industries, especially in the form of treating age-related disorders. Cellular senescence and loss of proliferative capacity along with critically shortened telomeres and dysfunctional telomerase activity has also been linked to multiple age-related diseases and aging syndromes including: idiopathic pulmonary fibrosis, bone marrow failure and age-related macular degeneration [9, 51]. These diseases could be treated or their effects lessened by extending telomeres and elevating proliferative capacity providing an additional potential use for LRP/LR. In conclusion, these findings illustrate a novel function of LRP/LR and telomerase in the process of cellular aging. Furthermore we show that with LRP::FLAG overexpression, it is possible to delay the accumulation of DNA damage or possibly repair it. Therefore, strategies aimed at elevating LRP/LR levels may prove a potential therapeutic treatment in reverting/impeding cellular senescence and, in turn organism aging.

## MATERIALS AND METHODS

### Cell lines

Non-tumorigenic, Human Embryonic Kidney cells (HEK293) obtained from ATCC were used due to their detectable levels of telomerase activity. Human lung fibroblasts (MRC 5) (Fox Chase Cancer Center) were used as the aging model due to low/undetectable levels of TERT and limited replications before reaching senescence. All cell lines were cultured in 5% CO_2_ at 37°C and were maintained in Dulbecco’s modified Eagle’s medium (DMEM) with high glucose (4.5g/l) (Hyclone) containing 4mM L-Glutamine, 10% foetal bovine serum (FBS) (Biowest) and 1% penicillin/streptomycin (Hyclone)(Thermo scientific).

### LRP::FLAG overexpression in HEK293 and MRC 5 cells

Cells were stably transfected to induce an overexpression of the LRP::FLAG protein, to elevate total levels of LRP within the cell [23]. Cells were transfected at a 60% confluency using Lipofectamine 3000 (Invitrogen), according to the manufacturer’s protocol. Briefly, 250μl Optimem, 5000ng of the pCIneo-moLRP-FLAG, 56.1μl P3000 and 56.1μl of the Lipofectamine reagent were mixed and incubated at room temperature for 10-15 minutes. Cells were treated with 250μl of the mix and incubated for 48 hours. Two days post treatment cells were exposed to the selective treatment, geneticin at 800ng/ml for 1 week. Thereafter, cells were maintained in 300ng/ml of geneticin to ensure transfection stability. In order to confirm success of the transfection, western blotting was carried out targeting the FLAG tag.

### Western blotting

Western blotting was used to determine the protein levels of LRP::FLAG, LRP/LR, hTERT and γH2AX post-transfection with pCIneo-moLRP-FLAG when compared to untreated controls. β-actin used as the loading control. Briefly, cells were lysed, protein levels quantified and 10μg of cell lysate was loaded for LRP/LR, and LRP::FLAG while 50μg was used for TERT and γH2AX. To ensure equal concentrations of lysate were used, β-actin was used as a loading control. These cell lysates were resolved on a SDS polyacrylamide gel and subsequently transferred to a polyvinylidene fluoride (PVDF) membrane using a semi-dry transferring apparatus at 350 mV for 50 minutes. Membranes were then blocked in 3% BSA in 1x PBS Tween (0.1%). Thereafter the appropriate primary antibody was added (see Supplementary Table 1 online) and incubated at 4°C overnight, with gentle agitation. Membranes were then washed in PBS-Tween, and further incubated in a suitably diluted solution of secondary antibody coupled to a HRP enzyme in 3% BSA in PBS-Tween for 1 hour at room temperature (see Supplementary Table 1 online). The membrane was washed as before and incubated with Biorad Clarity^™^ Western ECL substrate prior to being analyzed with the Biorad Gel Doc XR Imager.

### Confocal microscopy

Firstly, cells were seeded onto coverslips and cultured until 70% cell confluency was reached. The cells were fixed in a solution of 4% paraformaldehyde for 20 minutes, followed by three washes in 1X PBS. Cells were further permeabilized in a solution of 0.1% Triton X in PBS for 15 minutes. Thereafter, coverslips were washed thrice and blocked in 0.5% BSA for 20 minutes. The cells were subsequently incubated overnight with the primary antibodies for LRP/LR (1:100) and hTERT [(1:100) (see Supplementary Table 1 online)] and washed thrice with PBS. The cells were then incubated with the secondary antibodies [(1:100 dilution in 0.5% BSA) (see Supplementary Table 1 online)] for 1 hour where after the coverslips were washed and incubated for 10 minutes with 10μl of Dapi (10μg/ml) for nuclear staining. This was followed by additional washes. The cover slips were mounted and viewed at 400x and 630x magnification using the Zeiss LSM 780 confocal microscope.

### β-Galactosidase assays

An altered form Lee et al., 2006 [29] of the β-Galactosidase Enzyme Assay System with Reporter Lysis Buffer (Promega) was used to determine endogenous levels of β-Galactosidase within the cells. Briefly, cells were seeded into 6 well dishes and allowed to proliferate until confluent with daily media changes performed to prevent metabolic or stress related activation of β-galactosidase. The cells were harvested, washed in PBS and lysed with a lysis buffer provided in the kit. Lysates were quantified and standardized and thereafter, incubated in 96 well plates with Assay buffer composed of: sodium phosphate, MgCl_2_, and ortho-nitrophenyl-β-D-galactopyranoside (ONPG) all of which Lee et al., (2006) used for their soluble β-galactosidase assay. This mixture was then incubated at 37°C and allowed to react for sixteen hours. Negative controls consisting of lysis buffer and assay buffer only were also included. Afterwards sodium carbonate was added to stop the reaction and the plate was read at 420nm in a Sunrise^™^ ELISA reader (Tecan). The procedure was performed in triplicate with three biological repeats for both transfected and non-transfected HEK293 and MRC 5 cells.

### Detection of telomerase activity

Relative telomerase activity was quantified by the TRAPeze RT Telomerase detection Kit (Merck Millipore) following the manufacturers protocols with minor alterations. Briefly, cells were harvested and washed in PBS, after which they were lysed using CHAPS lysis buffer ((3-((3-cholamidopropyl) dimethylammonio)-1-propanesulfonate)). Protein and RNA extractions were collected in the supernatant where protein concentrations were standardized to 500 ng/μl for all experimental and control reactions. All samples were then subjected to experimental analysis by qPCR accompanied by a positive control (cell extract with confirmed telomerase activity) and three negative controls: no template control (water), CHAPs lysis buffer and samples heat treated at 85°C for 10 minutes. All reactions were performed in triplicate. The qPCR reactions were carried out in the LightCycler LC480 (Roche) using the following cycling parameters: 37°C for 30 minutes, 95°C for 2 minutes and 45 cycles of 95°C for 15 seconds, 59°C for 60 seconds and 45°C for 10 seconds. Telomerase activity was estimated by extrapolation from the standard curve generated by 1:10 serial dilutions (20–0.2 amoles) of TSR8 control as per Merck Millipore instructions. The data was analyzed with LightCycler1 Software version 1.5.1.

### Assessment of telomere length

The relative telomere length was assessed by use of Tel 1 and Tel 2 primers with qPCR as described by Cawthon *et al.* (2002). DNA was extracted using the Gene Jet DNA extraction kit (Thermo Scientific) following the manufacturer’s instructions. In order to normalize the data obtained for telomere length and to ensure accuracy in the quantification of the data obtained, 36B4 was used as a reference gene (List of primers used can be found as online Supplementary Table 2.) [28]. A standard curve was constructed using DNA of a known concentration (35ng/μl to 0.035ng/μl). The reaction was run with a set of positive (DNA samples previously confirmed to amplify) and negative controls (no template control). All experimental and control sample reactions were performed in the Roche LightCycler 480 (Germany) with the following cycling parameters: initial denaturation at 95°C for 10 minutes followed by 45 amplification cycles of: 95°C for 10 seconds, 58°C for 10 seconds and 72°C for 60 seconds. The amplification readings for the experimental samples were then normalized to the standard curve and analyzed.

### Statistical evaluations

All statistical evaluations of the data were carried out using Microsoft Excel 2014 (Microsoft Corporation) and Graph Prism Version 5 (Graphpad Software, Inc). All experiments were performed in at least triplicate so that standard deviation could be calculated. The two-tailed Student’s *t-*test was performed at a 95% confidence interval; where values *p<0.05 were considered significant, while values **p<0,01 and values ***p<0,001 were considered highly significant.

## SUPPLEMENTARY MATERIALS FIGURE AND TABLES



## Abbreviations

36B4: acidic ribosomal phosphoprotein
CHAPS: 3-((3-cholamidopropyl) dimethylammonio) 1-propanesulfonate
DAPI: 4’,6-diamidino-2-phenylindole
DDR: DNA Damage Response
DMEM: Dulbecco’s Modified Eagle’s Medium
DMSO: Dimethyl Sulfoxide
DNA: Deoxyribonucleic Acid
HEK293: Human Embryonic Kidney Cells
hTERT: Human Telomerase Reverse Transcriptase
kDa: Kilo Dalton
LRP/LR: 37 kDa Laminin Receptor Precursor/67 kDa High Affinity Laminin Receptor
MDA-MB231: Metastatic breast cancer cells
MRC-5: Human Lung Fibroblast cells
ONPG: Ortho-nitrophenyl-β-D-galactopyranoside
PAGE: Polyacrylamide Gel electrophoresis
PCR: Polymerase Chain Reaction
RNA: Ribonucleic Acid
TERC: Telomerase RNA Component
TERT: Telomerase Reverse Transcriptase
