# Supplementary material for: 37 kDa LRP::FLAG enhances telomerase activity and reduces senescent markers in vitro
Source: Oncotarget. 2017 Sep 27;8(49):86646–56. doi: 10.18632/oncotarget.21278 (PMC5689714; doi:10.18632/oncotarget.21278)
Supplement: Supplementary file 1 [file oncotarget-08-86646-s001.pdf]

37 kDa LRP::FLAG enhances telomerase activity and reduces senescent markers *in vitro*

SUPPLEMENTARY MATERIALS

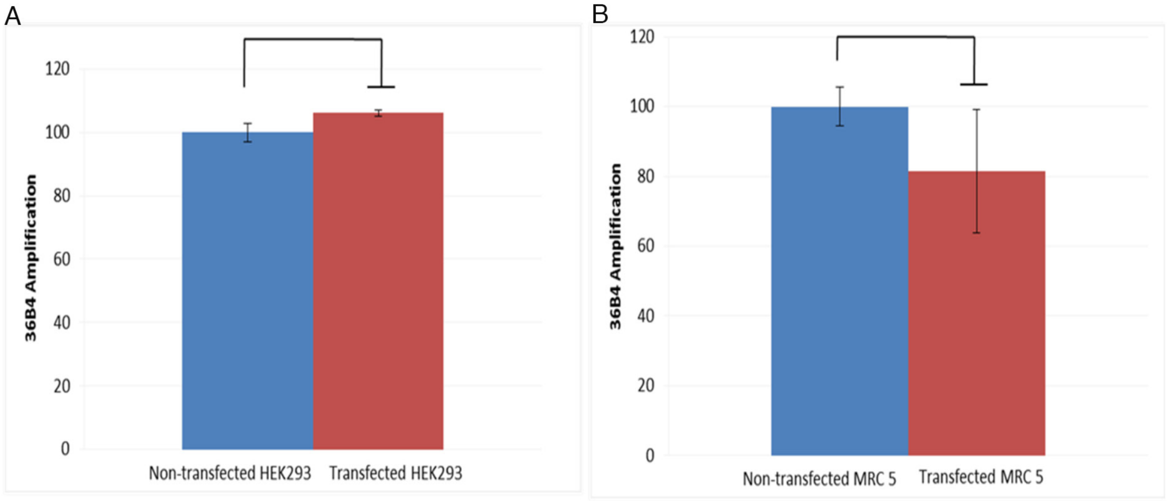

Supplementary Figure 1: qPCR amplification of reference gene 36B4. qPCR of the reference gene was performed to ensure that amplification levels between transfected and non-transfected HEK293 **(A)** and MRC 5 **(B)** samples were equivalent. Data analysis confirmed that there was no significant difference between transfected and non-transfected samples (HEK293: n= 3; p= 0.5405 and MRC 5: n= 3; p=0.054).

Supplementary Table 1: List of primary and secondary antibodies with concentrations for Western blotting, and Confocal microscopy

Target Protein	Primary antibody	Secondary antibody	Experiment	Dilution factor for both antibodies
LRP/LR	Human anti-LRP/LR IgG-iS18 (Affimed)	anti-human IgG-HRP (Abcam 6858)	Western blotting	1:6500
LRP/LR	Human anti-LRP/LR IgG-iS18 (Affimed)	anti-human IgG-FITC (Abcam 6854)	Confocal Microscopy	1:100
LRP::FLAG	Murine anti-FLAG (Sigma F-3165)	anti-murine IgG-HRP (Sigma A4416)	Western blotting	1:4000
LRP::FLAG	Murine anti-FLAG (Sigma F-3165)	anti-murine IgG-FITC (Abcam 6785)	Confocal Microscopy	1:100
hTERT	Rabbit anti-hTERT (abcam 183105)	anti-rabbit IgG-HRP (Cell signalling 7074S)	Western blotting	1:1000
hTERT	Rabbit anti-hTERT (Abcam 183105)	anti-rabbit IgG-APC (Abcam 72567)	Confocal Microscopy	1:100
β-actin	Murine anti-β-actin-peroxidase. (Sigma A3854)	-	Western blotting	1:10 000
γH2AX	Rabbit anti-Phospho-γH2AFX (PSER139) (Sigma SAB4300213)	anti-rabbit IgG-HRP (Cell signalling 7074S)	Western blotting	1:1000

Supplementary Table 2: List of primers utilised for all telomere length PCR related procedures

Primer name	5'- 3' sequence	Amplified region	Reference
Telomere length	TCCCGACTATCCCTATCCCTATCCCTATCCCTATCCCTA (Forward-Tel 1) GGTTTTTTGAGGGTGAGGGTGAGGGTGAGGGTGAGGGT (Reverse- Tel 2)	End of telomere	[25]
Reference gene: 36B4	CAGCAAGTGGGAAGGTGTAATCC (Forward) CCCATTCTATCATCAACGGGTACAA (Reverse)	36B4	[25]
